# Myocardial Damage and Inflammatory Response After Cardiac Surgical Revascularization on Beating and Arrested Heart

**DOI:** 10.21470/1678-9741-2024-0152

**Published:** 2025-11-17

**Authors:** Ante Bosnjak, Igor Rudez, Gordan Galic, Hrvoje Mikulic, Miro Mandic, Josko Petricevic

**Affiliations:** 1 Department of Cardiac Surgery, University Clinical Hospital Mostar, Mostar, Bosnia and Herzegovina; 2 Department of Cardiac and Transplant Surgery, Dubrava Clinical Hospital, Zagreb, Zagreb, Croatia; 3 Department of Pathology, Citology and Forensic Medicine, University Clinical Hospital Mostar, Bosnia and Herzegovina

**Keywords:** Coronary Artery Disease, Coronary Artery Bypass, Myocardial Revascularization, Systemic Inflammatory Response Syndrome.

## Abstract

**Introduction:**

Coronary artery bypass grafting remains the preferred method for surgical myocardial revascularization. The use of extracorporeal circulation during surgery has been linked to myocardial damage and a systemic inflammatory response. To mitigate these adverse effects, off-pump coronary artery bypass grafting was introduced as an effective and safe alternative. However, the comparison between these two procedures has yielded ambiguous results. The aim of our study was to determine the differences in myocardial damage and the intensity of the inflammatory response by measuring concentrations of troponin, cardiac isoenzyme of creatine kinase, leukocytes, and C-reactive protein at multiple time points within the first 24 hours postoperatively.

**Methods:**

This single-center, prospective study involved 61 patients diagnosed with coronary artery disease and divided into two groups based on the type of surgery performed.

**Results:**

Our results indicated that coronary artery bypass grafting with extracorporeal circulation is associated with greater myocardial damage, as evidenced by higher levels of troponin and cardiac isoenzyme of creatine kinase. Additionally, extracorporeal circulation was linked to a more pronounced increase in leukocyte count postoperatively. Unexpectedly, C-reactive protein levels were higher in the off-pump coronary artery bypass grafting group. There were no significant differences in hospital stay or in-hospital mortality between the two groups.

**Conclusion:**

Further research is necessary to clarify these controversies regarding the differences in systemic inflammatory responses between the two surgical approaches.

## INTRODUCTION

**Table:** 

Abbreviations, Acronyms & Symbols	
CABG	= Coronary artery bypass grafting
CHD	= Coronary heart disease
CK-MB	= Cardiac isoenzyme of creatine kinase
COPD	= Chronic obstructive pulmonary disease
CRP	= C-reactive protein
ECC	= Extracorporeal circulation
M	= Median
OPCAB	= Off-pump coronary artery bypass grafting
SD	= Standard deviation

The surgical treatment of ischemic coronary heart disease (CHD) without the use of extracorporeal circulation (ECC) devices theoretically represents an optimal modality for addressing coronary artery disease. Over the past three decades, the safety and comparable success rates of off-pump surgeries have been established, yet numerous studies comparing off-pump and on-pump coronary artery bypass grafting (CABG) have not conclusively demonstrated the superiority of the off-pump approach^[1-4]^. Certain patient populations, particularly those with pre-existing lung and/or kidney disease, exhibit a definitive benefit from avoiding ECC. These patients experience a lower incidence of postoperative respiratory and renal complications when ECC is not utilized^[5]^. Research indicates that the use of ECC is associated with a higher mortality rate during postoperative recovery due to renal complications^[1]^. Additionally, operating on a beating heart has shown advantages in patients over 70 years of age and those with significant left ventricular dysfunction^[2]^.

A study conducted in India highlighted the sensitivity of the coagulation system to ECC, demonstrating significantly higher activation of coagulation and fibrinolysis when ECC is employed^[6]^. This results in more pronounced hemolysis and fibrinolysis, leading to elevated blood nitrate levels postoperatively and adversely affecting renal and intestinal function. Visceral protection is better achieved when bypasses are performed without ECC^[5]^.

### Hemodynamic Stability and Complications

The primary disadvantage of performing CABG on a beating heart is the potential for hemodynamic instability during surgery, including a higher tendency for rhythm disturbances and the potential need for urgent conversion to ECC. Conversion typically requires a period of hemodynamic instability until ECC can adequately support tissue perfusion, with hypotension, hypoperfusion, and tissue hypoxia occurring until machine support is fully established. The duration of these adverse metabolic conditions largely depends on the surgeon's experience and skill, which can impact cerebral and visceral functional outcomes during and after surgery. However, careful patient selection can mitigate these issues, optimizing surgical outcomes by avoiding ECC-related complications.

### Inflammatory Response and Myocardial Damage

A study at Dubrava Clinical Hospital, Zagreb, revealed that surgeries on arrested hearts resulted in elevated levels of vascular inflammatory markers, such as endothelin-1 and troponin, compared to preoperative levels^[7]^. Conversely, in patients undergoing off-pump CABG (OPCAB), endothelin-1 and troponin levels remained stable postoperatively^[8]^. Other inflammatory markers, including interleukin 6, interleukin 8, and neopterin, also showed elevated serum levels when surgery was performed with ECC^[9]^. However, a conflicting study from Duke University in the United States of America reported higher postoperative troponin levels in patients undergoing OPCAB^[10]^. These discrepancies underscore the need for further research to establish the true comparative advantages of off-pump *vs.* on-pump CABG.

The aim of our study was to determine the differences in myocardial damage and the intensity of the inflammatory response by measuring concentrations of troponin, cardiac isoenzyme of creatine kinase (CK-MB), leukocytes, and C-reactive protein (CRP) at multiple time points within the first 24 hours postoperatively.

## METHODS

### Location and Time of Study

A prospective study was conducted at the Department of Cardiac Surgery of the University Clinical Hospital Mostar from January 2018 to January 2020. The study was approved by the University of Mostar Medical School Ethics Committee (approval number.: 01-I-1641-a/17).

### Participants

The study included 61 patients diagnosed with CHD who were indicated for cardiac surgery and aortocoronary bypass grafting following medical therapy, cardiology evaluation, and treatment.

### Inclusion Criteria

• Presence of CHD confirmed by coronary angiography.
• Elective surgery.
• Laboratory values of measured parameters within reference ranges before surgery (troponin, CK-MB, CRP, leukocytes).

Patients who met the inclusion criteria were informed about the nature of their disease, treatment options, and the aim of the research. Informed verbal consent was obtained from each patient.

### Exclusion Criteria

• Recent myocardial infarction.
• Patients with concurrent cardiac surgical disease.
• Chronic renal disease.
• Chronic lung disease.

### Measured Parameters in the Study

Measurements were taken from radial artery blood before the operation and at one, six, 12, and 24 hours postoperatively. Assessed parameters and their reference values are:

• Troponin: < 15.6 pg/ml
• CK-MB: < 24 U/L
• Leukocytes: from 3.5 to 10 × 10^9/L
• CRP: < 5 mg/dL

After surgery, patients were divided into two groups based on type of surgery. The first group (30 patients) underwent CABG with arrested heart and ECC, while the second group (31 patients) underwent OPCAB without ECC. The groups were compared based on differences in myocardial damage (troponin and CK-MB) and inflammatory response (leukocytes and CRP) at the specified time intervals.

### Rationale for Measured Parameters

• Troponin: A reliable marker of cardiac muscle trauma, rising within two - three hours post-injury, peaking at 24 hours, and persisting for one - two weeks.
• CK-MB: Although less specific than troponin, it historically served as an important marker for myocardial damage and is included here for comprehensive assessment.
• Leukocytes: Indicative of immune response, with increased counts reflecting the body's reaction to surgical trauma and ECC.
• CRP: An acute-phase protein synthesized in the liver, indicating tissue inflammation and stress response to surgical interventions, particularly involving ECC.

### Statistical Analysis

Data were collected using MS Excel (version 11. Microsoft Corporation, Redmond, Washington, United States of America) and analyzed with IBM Corp. Released 2012, IBM SPSS Statistics for Windows, version 21.0, Armonk, NY: IBM Corp. Descriptive statistics were used to present categorical variables as frequency and percentage, and continuous variables as mean and standard deviation (SD). For non-normally distributed data, the Chi-square test, Mann-Whitney U test, Friedman test, and Wilcoxon test were applied. For normally distributed data, two-way analysis of variance and Student's *t*-test for independent samples were used. A *P*-value of < 0.05 was considered statistically significant.

## RESULTS

### Descriptive Statistics

The study included 61 patients: 30 underwent surgery with an arrested heart (49.2%), and 31 underwent surgery on the beating heart (50.8%). Among the participants, nine were female (14.8%), and 52 were male (85.2%). The average patients’ age was 67.12 years (SD = 8.3, range 43-84). No significant difference was found in the average age of patients between the arrested heart group (median [M] = 68.37) and the beating heart group (M = 66.03) (t = 1.073, degrees of freedom = 56, *P* = 0.288). The sex distribution in each group is detailed in Table 1.

**Table 1:** Distribution of patients by sex according to type of surgery.

	CABG		OPCAB	
	n	%	n	%
Male	28	93.3	24	77.4
Female	2	6.7	7	22.6
Total	30	100.0	31	100.0

CABG=coronary artery bypass grafting; OPCAB=off-pump coronary artery bypass grafting

A significantly higher number of male patients was observed in both groups, with only two female patients undergoing surgery with an arrested heart. Due to the small number of female patients, sex differences were not statistically analyzed.

### Troponin

**Fig. 1:**
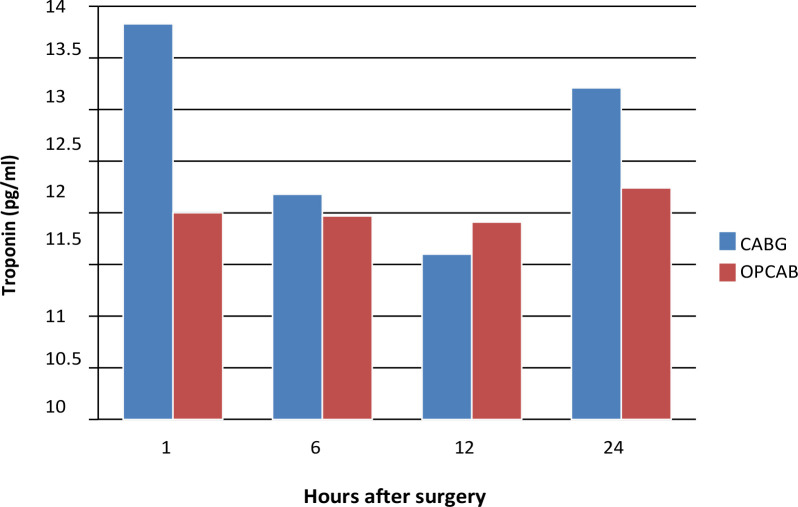
Troponin concentrations at one, six, 12, and 24 hours postoperatively in two groups. CABG=coronary artery bypass grafting; OPCAB=off-pump coronary artery bypass grafting.

Troponin concentrations increased gradually postoperatively in both groups, with consistently higher levels in the arrested heart group. This trend is depicted in Figure 1 and detailed in Table 2.

**Table 2:** Comparison of troponin parameter values between two groups of patients for each measurement point, using the Mann-Whitney U test.

	Surgery type	n	Average rank	Rank sum	Mann-Whitney U	z	*P*-value
Troponin 1 h	CABG	30	39.67	1190.00	205.00	-3.751	< 0.001^*^
	OPCAB	31	22.61	701.00			
Troponin 6 h	CABG	30	37.52	1125.50	269.50	-2.820	0.005^*^
	OPCAB	31	24.69	765.50			
Troponin 12 h	CABG	30	37.10	1113.00	282.00	-2.640	0.008^*^
	OPCAB	31	25.10	778.00			
Troponin 24 h	CABG	30	35.23	1057.00	338.00	-1.832	0.067
	OPCAB	31	26.90	834.00			

* *P* < 0.05. CABG=coronary artery bypass grafting; OPCAB=off-pump coronary artery bypass grafting

### Cardiac Isoenzyme of Creatine Kinase

**Fig. 2:**
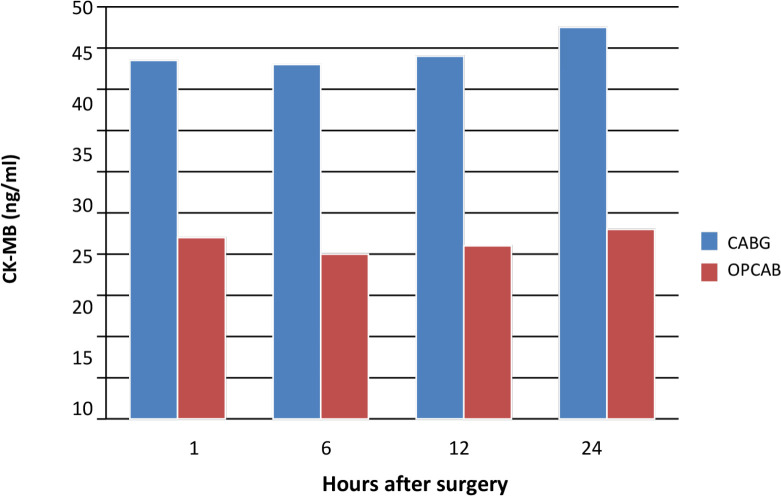
Cardiac isoenzyme of creatine kinase (CK-MB) concentrations at one, six, 12, and 24 hours postoperatively in two groups. CABG=coronary artery bypass grafting; OPCAB=off-pump coronary artery bypass grafting.

CK-MB concentrations decreased during the first six hours post-surgery in both groups. Subsequently, levels increased at 12 and 24 hours postoperatively, with higher overall values in the arrested heart group. These trends are shown in Figure 2 and detailed in Table 3.

**Table 3:** Comparison of CK-MB parameter values between two groups of patients for each measurement point, using the Mann-Whitney U test.

	Surgery type	n	Average rank	Rank sum	Mann- Whitney U	z	*P*-value
CK-MB 1 h	CABG	30	43.05	1291.50	103.50	-5.219	< 0.001
	OPCAB	31	19.34	599.50			
CK-MB 6 h	CABG	30	43.47	1304.00	91.00	-5.398	< 0.001
	OPCAB	31	18.94	587.00			
CK-MB 12 h	CABG	30	39.98	1199.50	195.50	-3.890	< 0.001
	OPCAB	31	22.31	691.50			
CK-MB 24 h	CABG	30	38.13	1144.00	251.00	-3.088	< 0.001
	OPCAB	31	24.10	747.00			

CABG=coronary artery bypass grafting; CK-MB=cardiac isoenzyme of creatine kinase; OPCAB=off-pump coronary artery bypass grafting

### Leukocytes

**Fig. 3:**
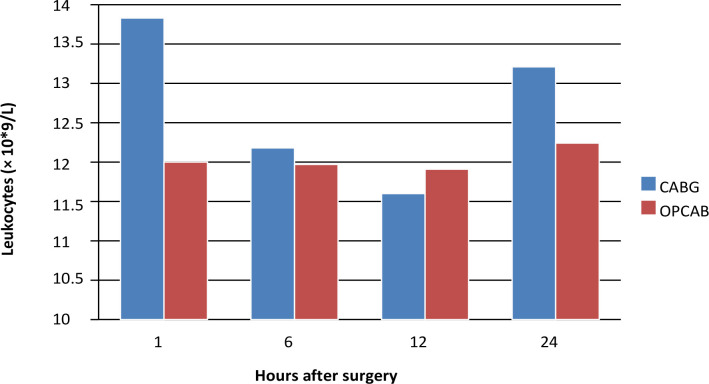
Average leukocyte concentration values at one, six, 12, and 24 hours postoperatively in two groups. CABG=coronary artery bypass grafting; OPCAB=off-pump coronary artery bypass grafting.

Leukocyte concentrations decreased during the first 12 hours post-surgery in both groups, followed by an increase in 24 hours. The increase in leukocyte concentration was lower in the beating heart group compared to the arrested heart group. This is illustrated in Figure 3 and detailed in Table 4.

**Table 4:** Comparison of the number of patients with normal or elevated leukocyte concentrations in both groups at individual measurement hours (n, %).

	CABG		OPCAB		χ2	*P*-value
	Normal	High	Normal	High		
1 h	7 (23.3%)	23 (76.7%)	10 (32.3%)	21 (67.7%)	0.604	0.437
6 h	7 (23.3%)	23 (76.7%)	10 (32.3%)	21 (67.7%)	0.604	0.437
12 h	8 (26.7%)	22 (73.3%)	10 (32.3%)	21 (67.7%)	0.229	0.632
24 h	6 (20%)	24 (80%)	8 (25.8%)	23 (74.2%)	0.291	0.590

CABG=coronary artery bypass grafting; OPCAB=off-pump coronary artery bypass grafting

### C-Reactive Protein

**Fig. 4:**
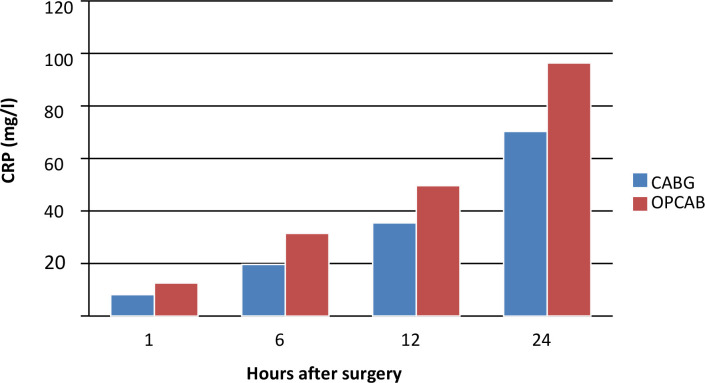
C-reactive protein (CRP) concentrations at one, six, 12, and 24 hours postoperatively in two groups. CABG=coronary artery bypass grafting; OPCAB=off-pump coronary artery bypass grafting.

CRP concentrations increased postoperatively in both groups, with higher levels observed in the beating heart group. This trend is shown in Figure 4 and Table 5.

**Table 5:** Average CRP concentration values at one, six, 12, and 24 hours postoperatively in two groups.

	CABG		OPCAB	
	M	SD	M	SD
CRP 1	8.13	10.408	12.56	17.056
CRP 6	19.63	13.908	31.50	37.500
CRP 12	35.46	18.738	49.67	38.758
CRP 24	70.33	23.494	96.38	45.220

CABG=coronary artery bypass grafting; CRP=C-reactive protein; M=median; OPCAB=off-pump coronary artery bypass grafting; SD=standard deviation

Although the increase in CRP values was statistically similar between the two groups, the beating heart group exhibited objectively higher CRP levels. CRP values at 12 and 24 hours postoperatively were elevated in all patients, with some within the reference range at one and six hours post-surgery. These details are presented in Table 6.

**Table 6:** Comparison of the number of patients with normal or elevated CRP concentrations in both groups at individual measurement hours (n, %).

	CABG		OPCAB		χ2	*P*-value
	Normal	High	Normal	High		
1 h	17 (56.7%)	13 (43.3%)	12 (38.7%)	19 (61.3%)	1.971	0.160
6 h	1 (3.3%)	29 (96.7%)	2 (6.5%)	29 (93.5%)		
12 h	0 (0%)	30 (100%)	0 (0%)	31 (100%)		
24 h	0 (0%)	30 (100%)	0 (0%)	31 (100%)		

CABG=coronary artery bypass grafting; CRP=C-reactive protein; OPCAB=off-pump coronary artery bypass grafting

## DISCUSSION

Cardiovascular diseases remain the leading cause of mortality worldwide, with CHD significantly impacting patient morbidity and mortality^[11]^. At our institution, it is estimated that 300 to 400 patients annually require surgical treatment for CHD. CABG constitutes two-thirds of all surgical procedures performed annually in our program. Therefore, it is crucial for cardiothoracic surgeons to understand the benefits and limitations of the current surgical techniques for treating coronary artery disease. This study was motivated by the need to objectively evaluate the impact of on-pump *vs.* off-pump CABG on myocardial injury and inflammatory response.

Our findings indicate that OPCAB results in less myocardial damage compared to on-pump CABG. This observation aligns with the study by Unić, Rudež, and colleagues at Dubrava Clinical Hospital in Zagreb, where elevated levels of endothelin-1 and troponin were found in patients undergoing on-pump surgery, signifying myocardial trauma. Conversely, endothelin-1 and troponin levels remained unchanged in off-pump patients^[7]^. However, a review of global studies reveals mixed results. While our findings favor OPCAB, other studies highlight the efficacy of on-pump CABG. For instance, The Danish On-pump Off-pump Randomisation Study (or DOORS) revealed a significantly higher rate of the primary composite outcome including all-cause mortality, repeat revascularization, or nonfatal myocardial infarction at one year and lower graft patency at six months following surgery in patients who underwent OPCAB^[4]^.

Regarding the inflammatory response, our study partially confirmed the hypothesis that CABG induces a stronger inflammatory reaction. Leukocyte counts were lower in the OPCAB group, consistent with expectations. Surprisingly, CRP levels were significantly higher postoperatively in the OPCAB group, a finding not entirely explained by the current literature. A study in United Kingdom associated higher CRP levels with longer hospital stays and recovery periods, indicating a need for further investigation into this phenomenon^[12]^.

### Limitations

The study's limitations include small sample size and inclusion of leukocyte count and CRP as the only measures of inflammatory response. Also, the exclusion of patients with significant comorbidities such as chronic obstructive pulmonary disease (COPD), renal insufficiency, recent myocardial infarction, and concomitant valvular heart disease. Consequently, the expected mortality rate, according to the European System for Cardiac Operative Risk Evaluation II, was 0.61%. This limitation restricts the generalizability of our findings to a broader patient population. Additionally, emergency patients were not included, which might have influenced the results. Future studies should focus on including patients with more severe comorbidities to determine the most appropriate surgical technique for different patient profiles.

## CONCLUSION

This study involved 61 patients with CHD who underwent cardiac revascularization and included several key findings. Induced cardiac arrest during surgery leads to greater myocardial damage. The use of ECC devices results in a more pronounced increase in leukocyte count postoperatively. Patients undergoing OPCAB exhibited a higher increase in CRP levels after surgery.

These results support the notion that OPCAB may play an increasingly significant role in the future. It is imperative for surgeons to be proficient in both techniques and to customize the surgical approach based on individual patient characteristics to optimize outcomes.

In my clinical experience, on-pump CABG is preferable for patients with slender, small-diameter coronary vessels due to the technical challenges they present. In contrast, OPCAB is advantageous for patients with comorbid conditions such as COPD or renal insufficiency. Ultimately, the choice of surgical technique should be tailored to the individual patient's condition and surgeon's expertise.

## Data Availability

The author declares that he does not have a special link for data repository but is ready to share it.

## Artificial Intelligence Usage

The authors declare use of ChatGPT for adjustment to academic writing in the preparation of this article.
